# 2D-fluoroscopic navigated percutaneous screw fixation of pelvic ring injuries - a case series

**DOI:** 10.1186/1471-2474-11-153

**Published:** 2010-07-07

**Authors:** Florian Gras, Ivan Marintschev, Arne Wilharm, Kajetan Klos, Thomas Mückley, Gunther O Hofmann

**Affiliations:** 1 Department of Trauma-, Hand- and Reconstructive Surgery Friedrich Schiller University Jena, Germany; 2 Berufsgenossenschaftliche Kliniken Halle, Germany

## Abstract

**Background:**

Screw fixation of pelvic ring fractures is a common, but demanding procedure and navigation techniques were introduced to increase the precision of screw placement. The purpose of this case series was the evaluation of screw misplacement rate and functional outcome of percutaneous screw fixation of pelvic ring disruptions using a 2D navigation system.

**Methods:**

Between August 2004 and December 2007, 44 of 442 patients with pelvic injuries were included for closed reduction and percutaneous screw fixation of disrupted pelvic ring lesions using an optoelectronic 2D-fluoroscopic based navigation system. Operating and fluoroscopy time were measured, as well as peri- and postoperative complications documented. Screw position was assessed by postoperative CT scans. Quality of live was evaluated by SF 36-questionnaire in 40 of 44 patients at mean follow up 15.5 ± 1.2 month.

**Results:**

56 iliosacral- and 29 ramus pubic-screws were inserted (mean operation time per screw 62 ± 4 minutes, mean fluoroscopy time per screw 123 ± 12 seconds). In post-operative CT-scans the screw position was assessed and graded as follows: I. secure positioning, completely in the cancellous bone (80%); II. secure positioning, but contacting cortical bone structures (14%); III. malplaced positioning, penetrating the cortical bone (6%). The malplacements predominantly occurred in bilateral overlapping screw fixation. No wound infection or iatrogenic neurovascular damage were observed. Four re-operations were performed, two of them due to implant-misplacement and two of them due to implant-failure.

**Conclusion:**

2D-fluoroscopic navigation is a safe tool providing high accuracy of percutaneous screw placement for pelvic ring fractures, but in cases of a bilateral iliosacral screw fixation an increased risk for screw misplacement was observed. If additional ramus pubic screw fixations are performed, the retrograde inserted screws have to pass the iliopubic eminence to prevent an axial screw loosening.

## Background

The goal of operative treatment for unstable pelvic ring fractures and sacroiliac dislocations is an anatomic reduction to restore the biomechanical axis and a rigid fixation to facilitate an early mobilization. Early operative treatment decreases immobilization-associated risks like thrombo-embolic events, pneumonia, decubital ulcer and psychological decompensation [1].

The current gold standard is an open or closed reduction and internal fixation [1-7]. Depending on the type of fracture and the localization, various approaches and fixation implants are established. For B1 as well as all C fractures (using the Tile classification system), stabilization of the anterior pelvic ring by a plate transfixation via the Pfannenstiel approach is the predominantly used technique [8]. The posterior pelvic ring is stabilized with different implants, such as internal fixators (vertebropelvic fixation), iliosacral screws or plates, ilioiliacal plates or Harrington sacral bars [2,3,5,6,9-16].

Aside from the severity of the initial trauma, postoperative complications such as soft tissue irritations and wound infections are approach related, most frequently in dorsal open reduction and stabilization techniques of the posterior pelvic ring [6,16].

Percutaneous iliosacral screw fixation procedures controlled by computed tomography (CT)- or fluoroscopic images are therefore increasingly used [7,9,14,17-21].

Due to the complex three-dimensional anatomy of the pelvis and the close proximity to neurovascular structures, the percutaneous screw fixation is demanding and requires the surgeon to have detailed anatomic knowledge and extensive surgical experience. In addition, the patient and the operating room team are exposed to high levels of fluoroscopy time [7,22].

To minimize these drawbacks, CT-based and later fluoroscopic-based navigation systems were introduced for screw fixation of pelvic ring injuries [15,21,23-26].

The aim of this case series was the evaluation of screw misplacement-rate and the functional outcome of percutaneous screw fixation of pelvic ring fractures and sacroiliac dislocations by using an optoelectronic navigation system based on 2D-fluoroscopic images.

## Methods

### Patient selection

Between August 2004 and December 2007, 442 patients with pelvic injuries (pelvic ring and acetabular fractures) were treated at our department. Stable A2 and partial stable Tile B2 fractures with moderate pain at mobilization were treated conservatively. 100 of 326 pelvic ring fractures were treated operatively. In 44 patients a navigated percutaneous screw fixation of the posterior pelvic ring was performed. Inclusion criteria were all B1, B3 and C fractures, in which a closed reduction could be achieved. B2 fractures were included in cases of intolerable pain at mobilization and patient refusal of stabilization with an external fixator or in cases of contra-lateral leg and foot injuries requiring none or partial weight bearing.

Exclusion criteria for 56 patients were crescent fractures, transiliac fractures, and comminuted transforaminal sacrum fractures.

24 patients had Tile B pelvic lesions (8 × B1, 14 × B2, 2 × B3) and 20 patients had Tile C pelvic lesions (13 × C1, 2 × C2, 5 × C3). Nineteen of them were females and 25 were males (total average age 36.7 years, range 16-70 years). All data were collected prospectively.

Eleven pelvic-lesions were initially stabilized in an emergency procedure, 9 by an external fixator and 2 by a pelvic clamp. In two cases due to persistent hemodynamic instability additional pelvic packing was necessary.

Two patients were treated immediately on the day of injury according to the concept of primary total care.

The overall mean Injury Severity Score (ISS) was 21 ± 9, the Hannover Polytrauma-Schlüssel (PTS) 24 ± 7.

Twenty one patients were polytraumatized (mean ISS 27 ± 2, mean PTS31 ± 3), 18 patients were multiple injured and five had an isolated pelvic ring injury. Causes of injury included 33 traffic accidents (17 by car, 2 by truck, 7 on a motor bike, 2 on a bicycle and 5 as pedestrians), 10 falls from a significant height and 1 industrial accident with crush injury. 59% of the patients were directly admitted to our department and 41% were initially treated at other hospitals and transferred to our unit for definitive pelvic surgery.

### Operation procedure

The operation was performed under general anesthesia by two experienced pelvic surgeons, after obtaining informed consent by the patient, and giving a detailed description of the innovative nature of the method and the possibility of an intraoperative switch to an open approach. The operations were carried out as soon as possible, based on patient clinical condition.

Preoperatively, the patients received urinary-catheters for reduction of bladder volume and when possible also a bowel enema for an unobstructed visualization of the bony pelvis in the fluoroscopic images. A single dose of antibiotic prophylaxis (1.5 g Cefuroxim i.v.) was given.

Cannulated partial thread screws (diameter: 6.5-8.0 mm, length 60-130 mm, ASNIS III, Stryker, Germany) were used as lag screws. The screws were placed over a 3.2 mm navigated guide wire. In cases of symphysis diastasis, these lesions were usually addressed first by plate-fixation (Matta Pelvic System, Stryker, Germany or Low Profile Pelvic System, Synthes, Germany). Depending on the type of fracture, SI-screws were placed in the first or second sacral vertebra (S1 or S2) as well as ramus pubic screws via a retrograde approach, as previously reported [3,11,14,22,27,28].

Patients were placed on a standard table (No.1150.30A0; Maquet, Germany) with a radiolucent carbon back plate and a plate extension (No.1150.45B0 and 1150.46B0; Maquet, Germany) in supine position with a sterile draped free moveable leg on the injured side to allow intraoperative manipulation for indirect reduction-maneuvers. External fixators or Schanz-screws were used as reduction aids (joysticks). The advantage of the supine position is the simultaneous reduction procedure of the anterior and posterior injuries. In 6 cases where a sufficient evaluation of the achieved reduction was not possible by 2D fluoroscopic images, an additional intraoperative 3D-fluoroscopic scan was performed, confirming an adequate reduction in each case (<5 millimeter dislocation). Otherwise a conversion to an open reduction would have been the next step in the protocol.

**Figure 1:**
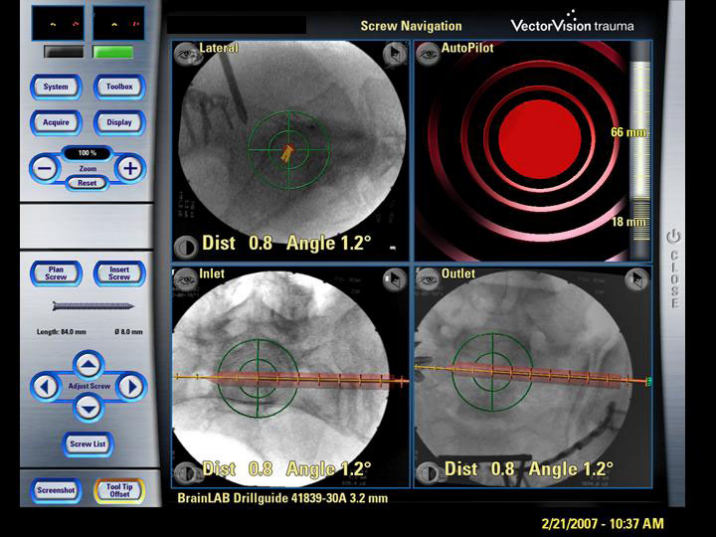
**Sterile covered touch screen of the navigation system (Vector Vision, Brainlab)**. displaying the standard images (lateral view, inlet, outlet) and an auto-pilot view. Red bar: virtually planned SI-screw; yellow line: prospective path of the navigated guide wire (trajectory), green bulls-eye: reflecting the exact positioning of navigated instruments to achieve the planned screw position.

Fixation of the minimal invasive array (MIA) via an additional skin incision on the opposite side of the intact pelvic ring (anterior iliac crest) was usually performed as the first operative step. Fluoroscopic images confirming the reduction in up to four different projections (depending on screw position: a.p., true lateral, inlet, outlet, ala inlet or obturator outlet) were obtained and transferred to the navigation system. The screw-position was planned on a sterile covered touch screen using these images (Fig. 1). After calibration of instruments and verification of their position in relation to the anatomic object, the navigation procedure was started. The entry point on the skin was determined by holding the navigated drill sleeve on the skin surface in the planned screw axis. Then a 2 cm skin incision and blunt preparation to the ilium were performed. During insertion, the pathway of the 3.2 mm guide wire through the different fluoroscopic planes was controlled virtually. Additional fluoroscopic images were only obtained before reaching the plane of the ipsilateral sacral neural foramina and at the final guide wire placement to control the position. The length of screws was measured by a reverse ruler. After drilling, the screws were fitted with a washer and placed with a cannulated screw driver.

### Image acquisition

**Figure 2:**
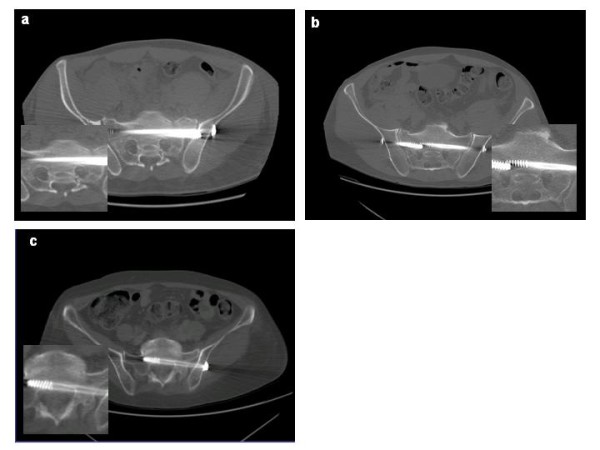
**CT-evaluation of screw position**. **a**: secure placement, completely in the cancellous bone; **b**: secure placement, but contacting cortical bone structures; **c**: malplacement, penetrating the cortical bone.

Pre- and postoperative CT scans (2.5 mm slices, Light Speed, General Electric, Germany) were performed for fracture classification and planning of the surgical procedure, as well as for postoperative evaluation of the reduction and screw placement (Fig. 2) [29]. The screw position was assessed using three categories:

I. secure placement, completely in the cancellous bone (Fig. 2a)

II. secure placement, but contacting the cortical bone (anterior sacrum, neural foramina or spinal canal) (Fig.2b)

III. malplacement, penetrating the cortical bone (Fig.2c).

### Navigation system

A 3.2 mm guiding sleeve was navigated based on 2D-fluoroscopic images (Ziehm Vario, Ziehm Imaging Inc., USA) using a passive optoelectronic navigation system (Vector Vision, Traumasoftware version 2.5.0 and version 2.6.1 since January 2007, Brainlab, Germany) (Fig.1). The technique of this procedure was described in detail by Kahler et al. and Stöckle et al. [11,15].

### Postoperative treatment and follow up examinations

Physiotherapy was started on the second postoperative day. Depending on the type of fracture, the patients were mobilized on crutches. Pain orientated full weight bearing was allowed immediately for Tile type B fractures, partial weight bearing (15 kg) for 6 weeks for all Tile type C fractures, except C3 fractures treated by bed rest for 3 weeks. Postoperatively, patients were seen for a physical examination at 6 weeks, 3, 6 and 12 months post-surgery and annually thereafter. At 6 weeks and 1 year follow up, radiological examinations and additional questionnaire evaluations using the SF-36 were administered (Tab.1) [30].

**Table 1:** SF-36-Scores at follow up of 15.2 ± 1.2 month

SF36-Subscores	PF	PR	BP	GH	VT	SF	ER	MH
**males < 35 (n = 12)**		*						

**Delta%**	-15.9	***-36.7***	-7.8	-15.0	-12.7	-8.9	-11.4	-11.5
**SD**	32.5	***59.9***	29.6	27.2	41.0	40.5	47.2	21.5
**SE**	9.4	***17.2***	8.6	7.9	11.8	11.7	13.6	6.2

**males **≥ **35 (n = 10)**								

**Delta%**	-2.0	-6.1	4.5	-3.8	18.0	14.1	10.9	1.9
**SD**	24.9	40.6	37.1	23.0	22.4	6.1	14.7	23.2
**SE**	7.9	12.9	11.7	7.3	7.1	1.9	4.6	7.3

**females < 35 (n = 9)**								
	
**Delta%**	-13.2	-3.3	-19.5	-18.9	-12.2	8.4	6.6	-14.4
**SD**	32.5	34.5	42.5	28.4	43.9	18.2	34.5	43.7
**SE**	10.8	11.5	14.2	9.5	14.6	6.1	11.5	14.6

**females **≥ **35 (n = 9)**								
	
**Delta%**	-15.5	-3.5	-10.7	-16.2	11.7	-1.1	-10.2	-2.5
**SD**	34.1	41.7	52.3	42.4	44.0	41.4	36.5	36.4
**SE**	11.4	13.9	17.4	14.1	14.7	13.8	12.2	12.1

Age groups < 35 und ≥35 years were combined for each gender to achieve a sufficient patient number per group for analysis. Values of the study group are compared to the normal age-correlated German population.

PF = Physical Functioning; PR = Physical Role; BP = Bodily Pain; GH = General Health; VT = Vitality, SF = Social Functioning; ER = Emotional Role; MH = Mental Health;

Mean Δ% = mean Delta in% of the healthy German population; SD = standard deviation, SE = standard error, [* = p < 0.05]

### Statistics

Results are expressed as means ± standard error. Differences were analyzed by a simultaneous comparison step-down-procedure of Bonferroni und Holm. P-values less than 0.05 were considered as significant. The study and follow up examinations were approved by the institutional ethics committee (No.2131-10/07).

## Results

In all cases reduction was achieved (<5 millimeters) [29]. None of the intended percutaneous procedures had to be cancelled due to poor visibility (obesity, excessive bowel air or contrast agents).

**Figure 3:**
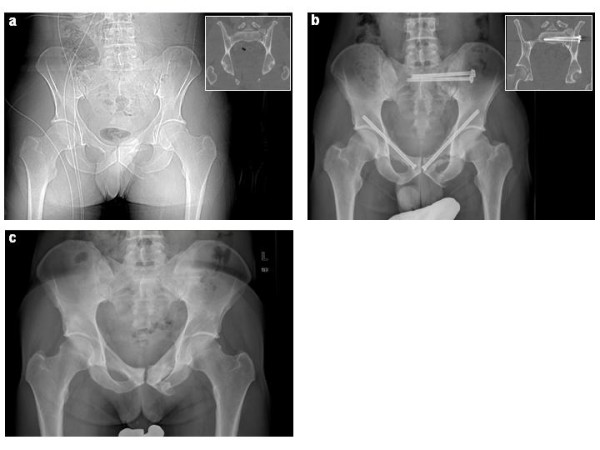
**36 y.o. male, motorcycle accident, pelvic fracture 61-C1.3.2c3**. **a**: initial posterior-anterior 2D CT-topography and coronal CT reformation (in inserted box) **b**: postoperative posterior-anterior x-ray and coronal CT reformation (in inserted box), **c**: posterior-anterior x-ray after implant removal

The operations were performed between the day of injury and the 23^rd ^day (mean 7 days) with a mean operating time of 163 ± 13 minutes (range 56 - 450 minutes), including approximately 10 minutes for fixation of the minimal invasive array and acquisition of the 2D-fluoroscopic images. The broad range of operating time is caused by a combined procedure of percutaneous screw and open plate fixation (12 cases with symphyseal diastasis) or simultaneous open fixation of acetabular fractures. The mean operating time of exclusively percutaneous pelvic ring fixations was 136 ± 9 minutes (62 ± 4 min/screw). Due to overlapping steps in the navigation algorithm (the once fixed reference array and some acquired 2D-fluororscopic images were used for more than one screw placement) only a pooled time for all screws inserted in one operation could be measured (Fig.3).

In total, we inserted 85 navigated screws for the fixation of pelvic ring injuries. 56 were placed iliosacral, 28 in the superior and one in the inferior pubic ramus. In five cases, two iliosacral screws were placed unilaterally in S1, in one patient one screw each was placed in S1 and S2, as well as in five patients a bilateral S1 fixation was performed (Fig.2b).

Mean fluoroscopy time per screw was 123 ± 12 seconds and included preoperative control of patients' position on the operating table, reduction maneuvers, image acquisition for the navigation procedure and intraoperative documentation of final screw position.

No screw misplacement was observed in intraoperative fluoroscopic control images, whereas in the postoperative CT scans 6% of the screws were malplaced, penetrating cortical bone structures (Fig.2c). In two cases, a screw was changed, due to anterior penetration and the fear of arrosion of the main vessels.

**Figure 4:**
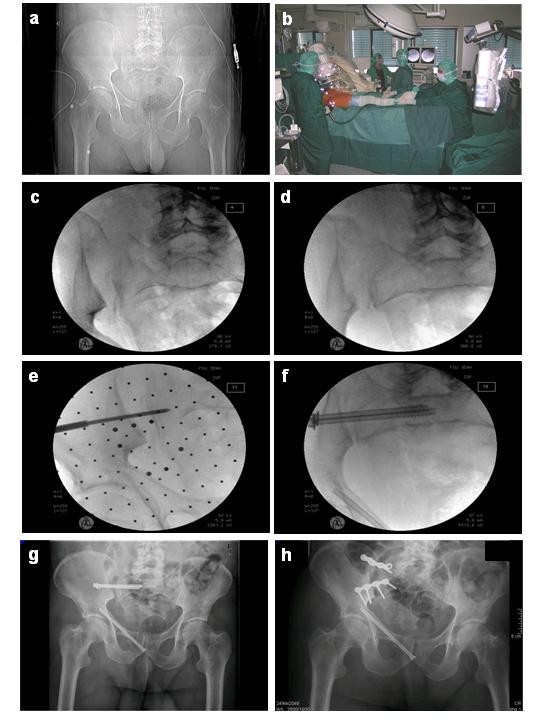
**63 y.o. male, 6 m fall from a tree, pelvic fracture 61-C1.2.1c1**. **a**: initial posterior-anterior 2D tomography, **b**: clinical photography of the operative set up with patient positioning and draping, reduction by traction on the leg and marking the skin incision with the navigated drill guide placed in the axis of the planned screw. **c + d**: intraoperative fluoroscopic images of the dislocation in posterior-anterior and inlet view, **e**: provisional fixation of the required height of ilium and sacrum with a k-wire, **f**: reduction and stabilization with two lag screws in the S1 body. **g**: postoperative loosening of the screws and re-dislocation of the SI joint **h**: despite operative revision with open reduction and anterior double plate fixation a new dislocation occurred.

In two other cases, an operative revision was required due to a secondary implant loosening during the hospital stay. In the first patient the fracture healed after replacing the loosened SI screw by two larger screws in S1. In the second patient (Fig.4), suffering of a left nephrectomy with normal serological and endocrine parameters, the operative fixation technique was changed. An anterior plate fixation of the sacroiliac joint was preferred in re-operation via an anterolateral approach. Nevertheless a fracture re-dislocation was observed 5 days after revision surgery. A second operative revision was refused by the patient. At the last follow up of 19 months, the patient was walking without crutches and satisfied with his health condition (SF-36: 120% for the physical component summary score; 86% for the mental component summary score compared to the age and gender controlled German population) [30].

One patient developed a prevesical haematoma, which was successfully treated by a percutaneous puncture. No iatrogenic neurovascular damage or perioperative complications such as disturbed wound healings, infections or thrombo-embolic events were observed. The overall post-operative hospital stay was 17 days (range 4-53 days).

40 of 44 patients (91%) were followed up at a mean of 15.5 ± 1.2 months. Of the other 4 patients, 1 had died of sequela of a severe head injury, 2 patients refused follow-up care and 1 patient, who was injured on journey, was lost for follow-up.

All except one pelvic ring fracture had healed at six months follow up x-rays. The one pseudarthrosis of a superior pubic ramus fracture was asymptomatic. In two cases a secondary axial loosening of ramus pubic screws were observed, followed by a subsequent implant removal in one case. No implant breakage was observed.

All questionnaires were filled out completely. For the quality of life no significant differences in the SF-36 Score were seen between the patients and the normal German population, except for the group of males <35 years old (Tab.1), whose physical role was significantly decreased (36.7 ± 17.2%, p < 0.05) [30].

## Discussion

### Operating- and fluoroscopy time

The first report of an open screw fixation of a sacroiliac dislocation was by Lehmann et al. (1934) [31]. Since then, the technique has undergone several modifications, based on Matta's widely established and standardized fluoroscopic projections (inlet and outlet) to evaluate the pelvic ring lesion and control the screw placement intraoperatively [2,14,32]. An additional true lateral view further increased the accuracy of transiliacal screw placements, especially for the screw placement in S2 [33].

Due to the limitations of 2D-fluoroscopic images, a repetitive change of the c-arm projection (90° to another) is mandatory to visualize the guide wire position in all three dimensions during drilling.

If a correction in one plane is necessary, a deviation in the third unobserved dimension can occur. In these situations, the guide wire has to be retracted and repositioned.

This procedure was described as "trial and error placing" by Kahler et al., resulting in high radiation exposure for the patient and the surgeon [11]. Even the most experienced surgeons reported mean fluoroscopy times of 126 seconds for placing iliosacral and 200 seconds for placing retrograde superior pubic ramus screws [4,17]. In some cases a mean fluoroscopy time of up to 18 minutes was needed for iliosacral and transiliacal screw positioning [22].

To avoid time-consuming changes of the c-arm position, the simultaneous use of two fluoroscopes was reported. This significantly reduces the fluoroscopy time (320 *vs. *270 seconds/screw), as well as operating time [7]. Disadvantages of this procedure are the need for two fluoroscopic units and limited space around the operating-table.

Using a 2D based navigation system, three fluoroscopic images have to be acquired once and then the movement of the guide wire could be visualized and virtually controlled on these images simultaneously. This technique reduces the fluoroscopy time and enables "first pass" guide wire placement reported in experimental settings so far [20,26]. In our clinical application we measured a fluoroscopic time of 123 ± 12 seconds per screw. Disadvantages of this technique are the virtual display of instrument position, which can differ with the real situation in cases of unnoted reference base movement (reference frame shift). Additionally a navigation independent intraosseous guide-wire bending can occur in a depth of more than 7.7 centimeters [34]. Therefore we recommend additional fluoroscopic images before reaching the neuroforamina, as well as in the final guide-wire position, before screw placement.

A clinical comparison of operation time for percutaneous screw insertion in different studies is difficult, due to the diverse nature and type of pelvic ring lesions (non- vs. displaced anterior or posterior pelvic ring fractures, post partum pelvic ring instability) and consequently different operating algorithms [11,23,25,35]. Furthermore the experience of the surgeon in pelvic surgery and in navigation procedures is an important factor, which may further influence these parameters.

As the experience of our team increased, an early definitive screw fixation was performed on the day of injury in two cases, instead of an initial fixation by a pelvic clamp or external fixator. The merits of early definitive operative treatment of pelvic fractures were recently discussed by Bircher et al. [36]. Especially closed reduction maneuvers should be performed as soon as possible to achieve optimal results.

### Accuracy of screw placement

In spite of a correct placement of screws in the intraoperative fluoroscopic control images, 6% of screw malplacements (perforation of the cortical bone) was seen in postoperative CT scans. This highlights the need for postoperative CT scans as a diagnostic tool to evaluate the screw placement [27,37,38]. In the literature malplacement rates for iliosacral screws are reported between 0 and 15% for the S1-position and the rate is even higher for the S2-position [15,20,23,26,35,37,39,40]. Besides the known anatomic risk factors:

I sacral dismorphism [33,41]

II malreduction [3,42] and

III iliosacral screws placed in S2 [17,24,37],

one additional risk factor was found in this study:

IV bilateral iliosacral screw fixation.

In these cases the exact placement of the contralateral screw is difficult, because of the potential guide wire deviation by the first inserted screw. A higher risk of screw misplacement or selecting shorter screws has to be considered.

Nevertheless, no iatrogenic operation-related damage of neurovascular structures were seen in this study, although serious complications such as violation of the superior gluteal artery and nerve roots have been reported[18,38,43,44].

Whether a 3D-fluoroscopic control scan should be recommended for intraoperative evaluation of definitive screw position, is still under discussion. Especially in one of our two cases needing a revision surgery after screw malplacement, two such 3D-scans would be required for complete imaging of the posterior pelvic ring, due to the limited field of view (maximum of 12 × 12 cm per 3D-fluoroscopic scan) [34]. Alternatively, intraoperative CT may solve this limitation, but due to high costs and demanding logistic requirements, this technology is available in a small number of trauma centers, only.

The use of recently introduced 3D fluoroscopy for navigation seems to further increase the accuracy of iliosacral screw placement compared to 2D fluoroscopy, as reported in two cadaver studies [20,26]. The CT like visualization of osseous structures and additional reformations, displaying orthogonal planes in front of the driller tip and offering a "view ahead", seems to be an advantage, but randomized clinical studies are warranted.

Evaluating the screw loosening of the anterior part of pelvic ring, both patients were female and the fracture line was in zone I of the Nakatani system[45]. According to these known risk factors for fixation failure described by the authors, we found an obvious insufficient length of the retrograde placed screws. After modification of our treatment standards, in which the thread part of the superior ramus pubic screw has to pass the iliopubic eminence completely, no further loosening of the last screws was seen.

An overall revision rate of 9% in this study is equivalent to the study of Schep et al., who treated 12 patients with postpartum pelvic pain by using a fluoroscopic navigation system for SI-screw placement [25].

However the use of this technique is limited to specialized trauma centers so far, because of the high costs and a long learning curve, as well as requirement of experience in standard open pelvic surgery.

## Conclusion

Our results confirm that 2D-fluoroscopic navigation is a safe tool providing a high accuracy of screw placement, but in cases of a bilateral iliosacral screw fixation an increased risk for screw misplacement was observed. For this fixation an intraoperative 3D fluoroscopic evaluation should be considered, if available. Additional retrograde ramus pubic screw fixation requires the pass of the iliopubic eminence by inserted screws to prevent an axial screw loosening.

## Competing interest

The authors declare that they have no competing interest.

## Authors' contributions

FG has made substantial contributions to design, reviewed the participants, analysed and summarized the data and drafted the manuscript. IM has made substantial contributions to design, performed the surgery, reviewed the participants and revised the manuscript. AW has made substantial contributions to acquisition of data. KK has made substantial contributions to acquisition of data. TM performed the surgery and has made substantial contributions to interpretation of data. GH identified the topic as an area of interest, reviewed and edited the manuscript. All authors have read and approved the final manuscript.

## Pre-publication history

The pre-publication history for this paper can be accessed here:

http://www.biomedcentral.com/1471-2474/11/153/prepub
